# Electro-physiology Models of Cells with Spherical Geometry with Non-conducting Center

**DOI:** 10.1007/s11538-020-00828-6

**Published:** 2020-11-19

**Authors:** Jiamu Jiang, Paul Smith, Mark C. W. van Rossum

**Affiliations:** 1 School of Mathematical Sciences, University of Nottingham, Nottingham, NG7 2RD UK; 2 School of Life Sciences, University of Nottingham, Nottingham, NG7 2RD UK; 3 School of Psychology, University of Nottingham, Nottingham, NG7 2RD UK

**Keywords:** Adipocytes, Ionic currents, Cable equation, Mathematical models

## Abstract

We study the flow of electrical currents in spherical cells with a non-conducting core, so that current flow is restricted to a thin shell below the cell’s membrane. Examples of such cells are fat storing cells (adipocytes). We derive the relation between current and voltage in the passive regime and examine the conditions under which the cell is electro-tonically compact. We compare our results to the well-studied case of electrical current flow in cylinder structures, such as neurons, described by the cable equation. In contrast to the cable, we find that for the sphere geometry (1) the voltage profile across the cell depends critically on the electrode geometry, and (2) the charging and discharging can be much faster than the membrane time constant; however, (3) voltage clamp experiments will incur similar distortion as in the cable case. We discuss the relevance for adipocyte function and experimental electro-physiology.

## Introduction

Many biological cells rely on electrical signals for intracellular and intercellular communication; this includes neurons but also other cell types, such as cardiac, muscle, and endocrine cells. Electrical currents have been most extensively studied in neurons. These have a tree-like geometry, and the branches of the tree are cylindrical structures with small diameters. Electrically, such cylinders are well modeled with the so-called cable equation (Jack et al. 1975; Koch 1999). The cable equation describes the spatio-temporal dynamics of the voltage along the cable in response to intracellular current injection along the cylinder.

The cable equation has been of great benefit to understand spatial-temporal integration in cells. For instance, it enables one to calculate how currents from distal synapses are filtered and contribute to the membrane voltage at arbitrary locations. Moreover, the cable equation is important for interpretation of experimental procedures, for instance to understand the fidelity of voltage clamp recordings. Finally, it can be extended to include active conductances to calculate the propagation of action potentials.

**Fig. 1:**
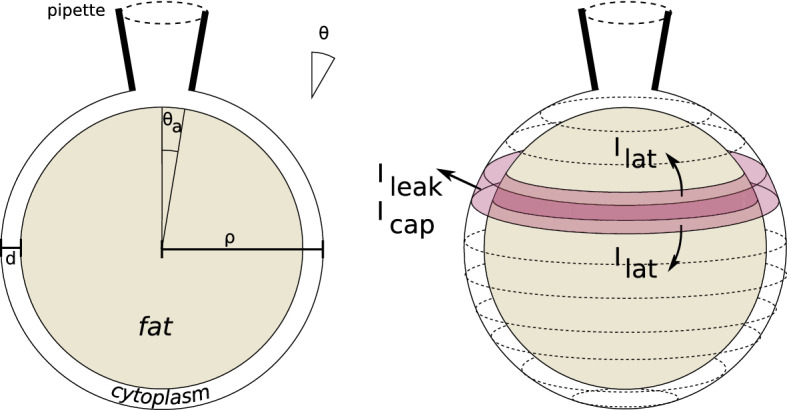
Left: The geometry under consideration. A spherical adipocyte with radius $\rho$ is largely filled with a fat globule; only the thin shell near the surface with thickness *d* conducts. Latitude on the sphere is indicated by the angle $\theta$. A pipette electrode with opening angle $\theta _{a}$ is attached at the top. Right: Construction to derive the sphere equation. The currents flowing in a given spherical segment at a certain latitude (pink) are indicated. The segment is assumed to be equipotential (Colour figure online)

The cable equation crucially relies on the effectively one dimensional geometry of the cylinder that it describes. However, some cells have very different geometries. Here, we in particular consider white fat adipocytes (Fig. 1). White fat adipocytes are the major store of energy and contain an intracellular lipid droplet, which is mobilized via lipolysis to release energy during times of calorific demand, while in time of calorific excess the lipid droplet is replenished (Arner et al. 2011). Adipocytes have a spherical geometry with a typical diameter of $\sim \,80\,\upmu$m, with 90–99% of the cell volume occupied by the unilocular lipid droplet (Thorsteinsson et al. 1976; Bentley et al. 2014). Consequently, most of the cytoplasm and the electric current flow is restricted to a limited intracellular space sandwiched between the lipid droplet and the plasma membrane of around $\sim \,0.5\,\upmu$m thickness (Carpentier et al. 1977; Cushman 1970; Williamson 1964).

Electrically, adipocytes have a resting membrane potential around − 30 mV, which is predominantly controlled by a passive membrane permeability to Cl$^{-}$ (Bentley et al. 2014). They also possess voltage-gated Ca$^{2+}$ channels that are spontaneously active at the relatively depolarized resting membrane potential with the associated Ca$^{2+}$ influx involved in the control of lipolysis (Fedorenko et al. 2020). Although generally considered electrically passive, the activity of voltage-gated channels in adipocytes can be modulated by hormones in response to the calorific demands of the body (Smith and Akaniro-Ejim 2020).

Without the insulating center of the fat droplet, the membrane voltage would be in very good approximation everywhere the same in the sphere. However, currents can only run in the thin spherical cytoplasmic shell between inner cell membrane and the fat (Fig. 1). Given this unique morphology, it is not clear how charges from localized ion fluxes spread across the cell (Fedorenko et al. 2020). Furthermore, accurate patch clamp recordings require the cell to be electro-tonically compact (Armstrong and Gilly 1992; Fedorenko et al. 2020), but the conditions for this are not known for the sphere geometry.

To answer such questions, we derive here the equivalent of the cable equation for spherical geometries and analyze its properties. In particular, we study the role of electrode geometry, the charging/discharging dynamics, and voltage clamp electro-physiology. The equivalent of the cable equation for a sphere geometry is not quite as trivial as might seem at first glance. First, once steady state has been reached in an infinite cable, the voltage will decay exponentially with distance from the injection site. However, in two (and higher)-dimensional geometries, the steady-state voltage response to a current modeled as a Dirac delta function in space diverges at the location of the injection, which is clearly unphysiological; thus, a more realistic model of the current injection is needed.

Secondly, for a cable increasing the distance between input and measurement site will decrease the response. However, how does this work on the sphere where increasing its radius will increase both the leak conductance but also reduces the intracellular resistance?

Finally, when a step current is applied to a single compartment, the equilibrium establishes according to an exponential charging curve with a time constant given by the membrane time constant. In long cables the charging can be substantially quicker, which is important when such experiments are used to determine biophysical parameters. However, the charging curve for sphere geometries is not known.

## The Sphere Equation

In analogy to the cable equation, we derive here the *sphere equation* that describes the spatial-temporal voltage on the sphere surface in response to a current injection. As we present our results largely by contrasting it to the cable equation, we first review the cable equation.

For the cable equation, one considers a cylindrical compartment of length *L* and small diameter $d_\mathrm{cable}$ ($d_\mathrm{cable}\ll L$). For typical neural geometries, one can ignore radial dependence in the voltage, so that the voltage is a function of position along the cable only (Rall 1969; Koch 1999). Although there are corrections on very brief timescales (Cartee and Plonsey 1992; Krassowska and Neu 1994; Lee and Grill 2005; Wang et al. 2018), we will ignore those here. The membrane separating the intracellular from the extra-cellular space has a specific capacitance $c_{m}$, and a specific membrane resistance $r_{m}$ from leak channels and channels open at rest. The membrane time constant is given by $\tau =r_{m}c_{m}$. The currents inside the cylinder experience an intracellular resistivity $r_{i}$.

Application of Kirchoff’s current law, which says that the sum of all currents is zero, leads to the 1D cable equation

$$\begin{aligned} \tau \frac{\partial V(x,t)}{\partial t}=-V(x,t)+\lambda _\mathrm{cable}^{2}\frac{\partial ^{2}V(x,t)}{\partial x^{2}}+I_\mathrm{ext}(x,t)r_{m} \end{aligned}$$

where *V*(*x*, *t*) is the intracellular voltage along the cylinder, and the electro-tonic length $\lambda _\mathrm{cable}=\sqrt{d_\mathrm{cable}r_{m}/4r_{i}}$ gives the typical spatial scale of voltage changes. Finally, $I_\mathrm{ext}$ represents the current density from the external stimuli, for instance from a synapse or experimental electrode. In the absence of active processes such as voltage-gated channels, the cable equation is linear and is mathematically equivalent to a 1D diffusion equation with absorption.

### Derivation of the Sphere Equation

To derive the equivalent equation on the sphere, we consider a sphere of radius $\rho$. Current flow is restricted to a thin shell of thickness *d* ($d\ll \rho$) below the surface (Fig. 1 left). The position on the sphere is given by the azimuth angle $\theta =0\ldots \pi$ and $\phi =0\ldots 2\pi$ (all angles will be in radians). We reorient the sphere so that the current injection site (typically from a patch pipette) is around the north pole. In that case the voltage is independent of $\phi$ and is described as $V(\theta ,t)$. To describe the evolution of the voltage, we discretize the angle $\theta$ in fine steps of size *h* (Fig. 1 right). In each spherical segment (a narrow band at given latitude), the voltage is assumed equipotential. We use an equivalent circuit model and apply Kirchoff’s equation, which state that the sum of the currents is zero in each segment, $0=\sum I=I_\mathrm{cap}+I_\mathrm{leak}+I_\mathrm{lat}+I_\mathrm{ext}$.

The capacitive current in a segment is given by $I_\mathrm{cap}=C_{m}\mathrm{d}V(\theta ,t)/\mathrm{d}t$, where the capacitance $C_{m}=c_{m}A$ is the product of the specific capacitance and *A* the outside area of the segment. Given the radius of the ball $\rho$, the area of the strip is $A=2\pi \rho ^{2}\left[ \cos (\theta -\frac{1}{2}h)-\cos (\theta +\frac{1}{2}h)\right]$, which in the limit of small *h* becomes $A=2\pi \rho ^{2}h\sin \theta$. For the leak, we assume that the current only leaks to the outside and that the outside potential is equipotential at zero. The leak current is then $I_\mathrm{leak}=V(\theta ,t)/R_{m}$, where $R_{m}=r_{m}/A$. One can introduce a leak reversal potential, or resting potential, *E*, so that $I_\mathrm{leak}=[V(\theta ,t)-E]/R_{m}$, this offsets all voltages by an amount *E*, but does not change anything else.

Finally, there will be currents coming from neighboring segments at other latitudes. These sum to $I_{lat}=[V(\theta -h,t)-V(\theta ,t)]/R_{i}^{-}+[V(\theta +h,t)-V(\theta ,t)]/R_{i}^{+}$, where $R_{i}^{\pm }$ is the total resistance between the bands at $\theta$ and $\theta \pm h$. This resistance is proportional to the distance between the centers of the segments, $\rho \sin h\approx \rho h$, and inversely proportional to the thickness of the shell *d* and the circumference at that latitude $S=2\pi \rho \sin (\theta \pm h/2)$. Hence $R_{i}^{\pm }=r_{i}h/[2\pi d\sin (\theta \pm h/2)]$ and $R_{m}/R_{i}^{\pm }=\frac{r_{m}}{r_{i}}\frac{d}{\rho ^{2}}\left[ \frac{1}{h^{2}}\pm \frac{1}{2h}\cot \theta \right]$. The biophysical parameters $r_{m}$ and $r_{i}$ are as in the cable, namely the specific membrane resistance and the intracellular resistivity, respectively.

Collecting all terms and taking the limit of small *h* so that the differences become spatial derivatives, we find

1 $$\begin{aligned} \tau \frac{\partial V(\theta ,t)}{\partial t}=-V(\theta ,t)+\frac{\lambda ^{2}}{\rho ^{2}}\frac{1}{\sin \theta }\frac{\partial }{\partial \theta }\left( \sin \theta \frac{\partial V(\theta ,t)}{\partial \theta }\right) +I_\mathrm{ext}(\theta ,t)R_{m} \end{aligned}$$

where

2 $$\begin{aligned} \lambda =\sqrt{r_{m}d/r_{i}} \end{aligned}$$

is a length scale. Note that apart from a geometrical factor 2 and the fact that *d* now describes the shell thickness instead of cable diameter, $\lambda$ is similar to the electro-tonic length in the cable equation.

Equation 1 is the “sphere equation”, the equivalent of the cable equation, describing the voltage on a spherical surface. It is a linear equation, with just two parameters: (1) the membrane time constant $\tau$ that describes how fast the voltage charges in response to local input currents, and (2) the ratio between $\lambda$ and radius $\rho$.

Just like the cable equation can be seen as a 1D diffusion equation, the sphere equation is a 3D diffusion equation in polar coordinates without radial and longitudinal fluxes ($\frac{\partial V}{\partial r}=\frac{\partial V}{\partial \phi }=0$). We assume that the external current and hence the voltage has no $\phi$ dependence. Inclusion of such terms would yield

$$\begin{aligned} \tau \frac{\partial V(\theta ,\phi ,t)}{\partial t}= & {} -V(\theta ,\phi ,t)+\frac{\lambda ^{2}}{\rho ^{2}}\frac{1}{\sin \theta }\frac{\partial }{\partial \theta }\left( \sin \theta \frac{\partial V(\theta ,\phi ,t)}{\partial \theta }\right) \\&\quad +\frac{\lambda ^{2}}{\rho ^{2}}\frac{1}{\sin ^{2}\theta }\frac{\partial ^{2}V(\theta ,\phi ,t)}{\partial \phi ^{2}}+I_\mathrm{ext}(\theta ,\phi ,t)R_{m} \end{aligned}$$

However, we shall restrict ourselves to Eq. 1, as this already allow us to study to most relevant and extreme cases.

The boundary condition

3 $$\begin{aligned} \frac{\partial V}{\partial \theta }|_{\theta =\pi }=0 \end{aligned}$$

ensures differentiability of the voltage at the south pole.

#### Modeling the Electrode

In the case of the cable equation, it is common to model input as Dirac delta functions. In the cable equation the voltage remains finite as one decreases the spatial extent of the input. However, as we will illustrate below, this is no longer true on the sphere. As the current is forced through a smaller and smaller electrode the local voltage diverges to infinity. Mathematically, this corresponds to the divergence of the Green’s function of the diffusion equation in 2 or more dimensions (Carslaw and Jaeger 1959).

To prevent this divergence, we model the finite size of the electrode. We assume that current is injected distributed over the surface $\theta =0\ldots \theta _{a}$ (Fig. 1). The area covered by the electrode ($\theta \le \theta _{a}$) has different properties: First, as the resistance within the electrode is low, the voltage under the electrode is assumed equipotential. Secondly, as there is no leak or capacitive coupling with the outside, all current injected via the electrode leaves as current at latitude $\theta _{a}$. The total amount of current there is $-I_\mathrm{ext}=\frac{1}{R_{i}}\lim _{h\rightarrow 0}V(\theta _{a}+h)-V(\theta _{a})$, or

4 $$\begin{aligned} V'(\theta _{a})=-I_\mathrm{ext}\frac{r_{i}}{2\pi d\sin \theta _{a}} \end{aligned}$$

Thus, the presence of the current injection electrode leads to an additional boundary condition at $\theta _{a}$.

In summary, the sphere equation is solved for $\theta _{a}\le \theta \le \pi$ with two boundary conditions; the voltage for $\theta \le \theta _{a}$ is assumed constant. Unless stated otherwise, we use $\theta _{a}=0.1$ for the figures.

### Steady-State Voltage Distribution

We are now in a position to solve the sphere equation. First, we consider the case that a steady current is injected, and the voltage has equilibrated. The steady-state voltage distribution on the sphere can be found by setting the left-hand side of Eq. 1 to zero.

The solution for the steady-state voltage are the Legendre functions $P_{\nu }(\cos \theta )$ and $Q_{\nu }(\cos \theta )$, where the parameter can take either root $\nu ^{\pm }=-\frac{1}{2}\pm \frac{1}{2}\sqrt{1-4\rho ^{2}/\lambda ^{2}}$ (Gradshteyn and Ryzhik 2014). In principle one thus has four solutions, but as $P_{\nu }(\cos \theta )=P_{-1-\nu }(\cos \theta )$ three independent solutions are left.

It is useful to mathematically analyze the case of real and complex roots of $\nu$ separately, although the biophysics behaves no differently in these regimes. For $\rho /\lambda \le 1/2$, $\nu ^{+}$ and $\nu ^{-}=1-\nu ^{+}$ are real and so are the Legendre functions. Both *P* and *Q* diverge at $\theta =\pi$. The boundary condition at the south pole can only be fulfilled by setting $V(\theta )=b\left[ Q_{\nu ^{+}}(\cos \theta )+Q_{\nu ^{-}}(\cos \theta )\right]$, and this cancels the divergence and ensures that $V'(\pi )=0$.

For $\rho /\lambda >1/2$, the roots become complex and *P* and *Q* are known as conical functions. While *P* remains real and divergent at $\theta =\pi$, *Q* no longer diverges but becomes complex. As $\mathfrak {I}(Q_{\nu ^{+}}(x))=-\mathfrak {I}(Q_{\nu ^{-}}(x))$, the real part is extracted through adding the solutions.

Thus, in both regimes the steady-state voltage is

5 $$\begin{aligned} V(\theta )=b\left[ Q_{\nu ^{+}}(\cos \theta )+Q_{\nu ^{-}}(\cos \theta )\right] \end{aligned}$$

The normalization constant

$$\begin{aligned} b=-\frac{I_\mathrm{ext}r_{i}}{2\pi d\sin ^{2}\theta _{a}}\frac{1}{Q'_{\nu ^{+}}(\cos \theta _{a})+Q'_{\nu ^{-}}(\cos \theta _{a})} \end{aligned}$$

follows from the boundary condition at the pipette (Eq. 4), where $Q'(z)=dQ(z)/dz$.

**Fig. 2:**
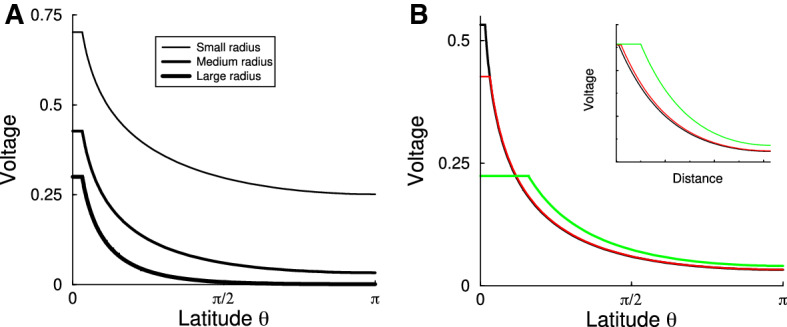
Steady-state voltage in response to a sustained current injection at the north pole. **a** Voltage for a sphere with small radius (top) to large radius. For the larger radius the voltage barely reaches the bottom of the sphere. Radius (from top to bottom) $\rho /\lambda =1/\sqrt{2},1,\sqrt{2}$. **b** Voltage profile in response to constant current injection for various pipette diameters. The narrower the pipette, the higher the voltage under the pipette, as the current needs to flow through a smaller hole. Pipette angle (bottom to top) $\theta _{a}=0.5,0.1,0.05$. (Radius $\rho =\lambda$). Inset: Effect of changing pipette size in a cable. In contrast to the sphere, the voltage excursion at the site of the pipette is virtually independent of the size in the pipette (cable length $L=\pi \lambda$) (Colour figure online)

The steady-state voltage is shown in Fig. 2a. The voltage excursion is linear in the stimulus current; hence, it is plotted in units of input current $I_\mathrm{ext}r_{i}/d$. The shape of the steady-state solution depends only on the ratio between the radius of the sphere $\rho$ and the spatial scale $\lambda$.

If $\rho \ll \lambda$ (Fig. 2a, top curve), the voltage becomes identical across the sphere, i.e., the sphere is electro-tonically compact, and the cell approaches the behavior of a single compartment. For a single compartment, the voltage is described by a resistor in parallel with a capacitor, so that $\tau \frac{\mathrm{d}V(t)}{\mathrm{d}t}=-V(t)+I_\mathrm{ext}$. On the other hand if $\rho \gg \lambda$ (bottom curve), the effect of the current injection remains localized to the injection site. In addition to reducing the diameter, increasing $r_{m}$ or *d*, or decreasing $r_{i}$ will make the sphere electro-tonically more compact (see Eq. 2).

The effect of the pipette diameter on steady-state voltage is shown in Fig. 2b. As the pipette becomes smaller, but with the same amount of current injected, the voltage excursion at the injection site becomes larger. For small $\theta _{a}$, the voltage at the pipette diverges with smaller pipette diameter as $V(\theta _{a})=-\frac{Ir_{i}}{2\pi d}\log \theta _{a}$. In comparison, in a cable there is no such divergence when the pipette diameter is made smaller, and the local voltage excursion is practically independent of the electrode size (Fig. 2b, inset).

As a result of this divergence, the input resistance, defined as $R_{in}=V/I$, also diverges with very small pipette diameters. This is the case even for electro-tonically nearly compact cells, although the size of the pipette where this behavior is noticeable becomes very small for typical adipocytes (see below). Of course, very high voltage gradients are problematic biologically. On very small spatial scales the sphere Eq. 1 is expected to break down—as any other diffusion-type equation—and a microscopic electro-diffusion approach will be needed (Qian and Sejnowski 1989).

Also when numerically solving the sphere equation, the divergence can lead to inaccuracy. One can check the accuracy of the steady-state solution, by realizing that all current provided by the external stimulus must find its way out as leak. Hence, $I_\mathrm{leak}=\frac{1}{r_{m}}\int _{\Omega }V(\theta )=\frac{2\pi \rho ^{2}}{r_{m}}\int _{\theta _{a}}^{\pi }V(\theta )\sin \theta \,d\theta$ should equal $I_\mathrm{ext}$. We used this identity to check whether the numerical solution found for $V(\theta )$ is accurate.

### Time-Dependent Solutions

To analyze the time-dependent solution of the sphere equation, we rely on numerical solution. We checked that this solution produced the correct steady-state solution derived above.

#### Filtering and Charging Time

**Fig. 3:**
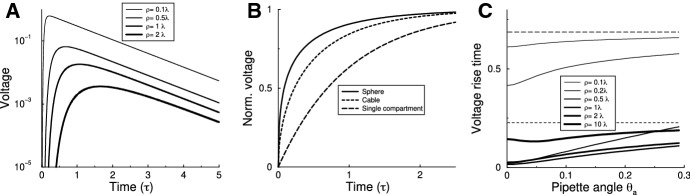
Voltage dynamics in the sphere for various experiments. **a** Voltage at the south pole in response to a brief current pulse at the north pole. A larger radius leads to smaller, more filtered responses. **b** Voltage evolution at the site of current injection in response to a step current, for a single compartment, an infinite cable, and a sphere (radius $\rho =\lambda$, $\theta _{a}=0.1$). Voltages are normalized to the final voltage. **c** Experiment as in panel. Time to reach half maximum voltage against pipette diameter for various sphere diameters. Shown for comparison: time to half maximum for a single compartment (dashed line) and cable (dotted line)

First, we inject a short current pulse through the pipette and measure the voltage response at the south pole. As expected, the voltage response rapidly rises, and then decays exponentially with the membrane time constant (Fig. 3a). The larger the sphere, the stronger the filtering. This is similar to what happens in a cable: far away inputs are filtered more.

Next, we consider how quickly the equilibrium is established when a step current is applied. When a step current is applied to a single compartment, the equilibrium voltage establishes according to an exponential charging curve with a time constant given by the membrane time constant $V=I_{0}\exp (-t/\tau )/R$, with an exponential time course with time constant $\tau =RC$. However, in the cable the voltage at the point of injection grows *faster*. This is easiest to understand by considering the equivalent case of *dis*-charging, where a constant current is suddenly removed and voltage decays. The intuition is that in a single compartment the charge only flows out through the membrane leak, but in the cable the charge decays faster because it can also flow laterally into the cable. In an infinite cable the voltage charging/discharging behaves as $V(x=0,t)\sim \mathrm {erf}(\sqrt{t/\tau })$ (Rall 1957; Jack et al. 1975). This well-studied effect is important when one uses the charging curves to extract the membrane time constant. When the cable is made shorter, the charging time will start to approach the single compartment result.

An example of charging in the sphere is shown in Fig. 3b, comparing it to a single compartment and an infinite cable. In this case the charging is faster than either single compartment or cable. In Fig. 3c we plot the time for the voltage to reach half of its final value. For comparison we indicated the charging time for the single compartment, $\tau \log 2\approx 0.693\tau$ (dashed line) and for the cable, $\tau erf^{(-1)} (1/2) \approx 0.227 \tau$ (dotted line).

The figure reveals a complex non-monotonic relation between charging time and sphere radius and pipette angle. However, some intuition can be obtained from limiting cases: When the sphere is very compact ($\rho /\lambda \ll 1$), the charging is similar to that of a single compartment and independent of the pipette angle (thin curves). Increasing the sphere diameter reduces the charging time. In the limit of a very narrow pipette the charging time goes to zero. However, in the limit when $\rho \theta _{a}\gg \lambda$, the voltage excursion is limited to a narrow latitude far from the north pole and thus the space over which the voltage spreads is approximately flat. Here one approaches the charging time found for the cable (thickest curve).

### Voltage Clamp

Finally, we consider a common experimental protocol to measure active currents in a cell: the voltage clamp method introduced by Cole (Hodgkin and Huxley 1952). In a voltage clamp experiment, the injection current is adjusted via a feedback circuit such that voltage at the injection site remains the same. In an ideal voltage clamp, there is no capacitive current (as $\mathrm{d}V/\mathrm{d}t=0$), and the current conservation law implies that the injected clamp current perfectly reflects the input currents to the cell without any filtering. It is well known that when the cell is not electro-tonically compact, the voltage at the input site is not fully controlled, and the clamp current is a filtered version of the input current (Armstrong and Gilly 1992; Williams and Mitchell 2008).

**Fig. 4:**
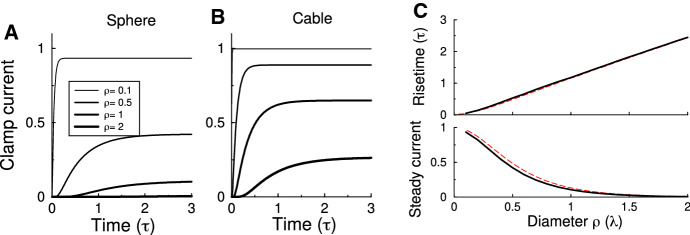
Current measurement using voltage clamp. **a** The clamp current measured at the north pole in response to a step current input at the south pole for spheres of different radii. Larger spheres lead to more filtering and larger “space-clamp” errors. **b** Voltage clamp errors for a cable of length $L/\lambda =\{0.1,0.5,1,2\}$. **c** The amplitude and half-maximum rise time of the clamp current across sphere radius. The filtering is similar to that in a cable of matched length (red-dashed curve) (Colour figure online)

To examine the feasibility of voltage clamp in the sphere geometry, we assume an electrode at the north pole and clamp its voltage at 0mV. A step current is injected at the south pole, and the clamp current measured for a variety of sphere sizes (Fig. 4a). Having the injection site opposite the measurement site maximizes the filtering, but also means that no longitudinal dependence is required in the model.

As expected, for small spheres, the clamp current reflects the input current well, but for larger spheres, there is a substantial reduction in the amplitude and strong temporal filtering. We further characterized the distortion by the amplitude reduction and the time to half-maximum (Fig. 4c). The distortion in the sphere is similar to that in a finite cable with input provided at the far end of the cable. Numerically agreement is achieved when the sphere diameter $\rho /\lambda \approx 2.7L/\lambda _\mathrm{cable}$ (red dashed curve). The results are virtually independent of the pipette diameter (not shown).

Thus, in summary in the sphere geometry, voltage clamp experiments are feasible and are subject to similar distortion as in the cable.

### Application to Adipocytes

What do these findings mean for adipocytes? Typical adipocyte has a radius $\rho =40\,\upmu$m, shell thickness $d=0.5\,\upmu$m (Williamson 1964; Cushman 1970; Carpentier et al. 1977). The other parameters are not well known. (Indeed, the analysis presented here should help their determination.) Using neural values one has specific capacitance $c_{m}=1\,\upmu \mathrm{F}/\mathrm{cm}^{2}$ (lipid bi-layer) and specific intracellular resistance $r_{i}=100\,\Omega \,\mathrm{cm}$ from ionic currents in cytoplasm. We use a specific membrane leak $r_{m}=100\,\mathrm{k}\Omega \mathrm{cm}^{2}$ (Bentley et al. 2014). This means that $\tau =100$ ms and that $\lambda \approx 2200\,\upmu$m, which is much larger than the radius; $\lambda /\rho \approx 55\gg 1$. Therefore, the adipocyte should be electro-tonically compact.

A typical pipette is about $2\,\upmu \hbox {m}$ in diameter, so that $\theta _{a}=0.025$. Using these values in Eq. 5 yields the voltage at the pipette and an input resistance of 481 $\mathrm{M}\Omega$. Comparing this to a single compartment model, we find that the input resistance is only slightly overestimated by 0.27%. Next, we repeated the analysis of Fig. 3c and find that the charging time to half maximum is 0.6919 times the membrane time constant, only slightly faster than would be expected from a single compartment.

## Discussion

In most spherical cells currents can flow through the center ensuring that the voltage is very similar across the cell. In this study we have considered electric current flow in a spherical geometry, such as adipocytes in which currents are restricted in a thin layer around its central fat globule. In this case we cannot assume the cell to be equipotential. For this geometry, we derived the sphere equation, an analogue of the cable equation widely used to study electric current flows in cylinder geometries. We find that the voltage across the membrane is characterized by two parameters: the time constant of the membrane $\tau$, and the ratio between the radius of the sphere and the constant $\lambda$. While the sphere equation—like the cable equation—is only valid for passive membranes without voltage-gated channels, inclusion of active conductances is in principle straightforward, but will typically require numerical solution.

Our analysis reveals differences and commonalities between the cable equation and the sphere equation. First, the spread of the voltage on the sphere in response to a current injection depends on how electro-tonically compact the sphere is, as in the cable. However, in contrast to the cable equation, the voltage at the electrode diverges as the pipette is made smaller. Thus, the measured voltages and input resistance will depend on pipette size.

Another difference is observed when charging/discharging the membrane voltage through a current injection. Here, we find that the charging time can be much faster than the membrane time constant and that it strongly depends on the electrode size. Hence, such experiments cannot be easily used to determine the membrane time constant. Lastly, we examined how the sphere geometry affects voltage clamp experiments. Here, we found that *mutatis mutandi* the distortions known in the cable equation, are the same in the sphere equation.

Finally, our analysis shows that for typical adipocyte experiments systematic but small deviations from the single compartment can be expected. Thus, while for the given biophysical properties of adipocytes and the typical pipette diameter, the corrections are small, our analysis allows to fully quantify such errors, also when the parameters change.

### Numerical Methods

The steady solution was analyzed using Mathematica, and the numerical solution of the time-dependent equation was obtained with MATLAB. Code is online at https://github.com/vanrossumlab/jiamu_20.
